# Antiviral immune response against HTLV-1 invalidates T-SPOT.TB^®^ results in patients with HTLV-1-positive rheumatic diseases

**DOI:** 10.3389/fimmu.2024.1480506

**Published:** 2024-10-29

**Authors:** Masatoshi Kimura, Kunihiko Umekita, Chihiro Iwao, Katsumi Kawano, Yuki Hashikura, Yayoi Hashiba, Toshihiko Hidaka, Kenji Sugata, Yorifumi Satou, Taiga Miyazaki

**Affiliations:** ^1^ Division of Respirology, Rheumatology, Infectious Diseases and Neurology, Department of Internal Medicine, University of Miyazaki, Miyazaki, Japan; ^2^ Clinical Laboratory, University of Miyazaki of Hospital, Miyazaki, Japan; ^3^ Institute of Rheumatology, Miyazaki Zenjinkai Hospital, Miyazaki, Japan; ^4^ Division of Genomics and Transcriptomics, Joint Research Center for Human Retrovirus Infection, Kumamoto University, Kumamoto, Japan

**Keywords:** HTLV-1, IFN-γ-releasing assay, rheumatic disease, latent tuberculosis infection, cytotoxic T lymphocytes

## Abstract

**Background:**

T-SPOT.TB^®^, one of the screening tests for latent tuberculosis infection (LTBI), yields invalid results in human T-cell leukemia virus type 1 (HTLV-1)-positive patients with rheumatoid arthritis. However, the detailed mechanisms behind this invalidation are unclear. Additionally, it remains unclear whether T-SPOT.TB^®^ or QuantiFERON-TB (QFT) is more useful in HTLV-1-positive patients with rheumatic disease (RD).

**Method:**

Among all of the HTLV-1-positive RD patients who visited our department between August 2012 and December 2022, 44 patients who were screened using T-SPOT.TB® were included in the analysis. QFT testing was performed in 33 of the 44 patients, and the results were compared with that of T-SPOT.TB®. Furthermore, we performed a culture experiment mimicking T-SPOT.TB® using peripheral blood mononuclear cells (PBMCs) obtained from HTLV-1-positive patients with RD. Additionally, T-cell subsets with autonomous product IFN-γ were analyzed using a flow cytometer.

**Results:**

Of the included patients, 13 (29.5%) were invalid for T-SPOT.TB® because of the increased number of negative control spots. The median HTLV-1 proviral load in the invalid group was higher than that in the valid group (2.45 vs. 0.49 copies/100 PBMCs, respectively, *p* = 0.002). QFT was performed in all 33 patients, including 13 patients who were invalid in T-SPOT.TB®. The main source of IFN-γ production was CD8+ T-cells in the T-SPOT.TB® mimic experiment. Furthermore, Tax-expressing CD4+ T-cells and Tax-specific cytotoxic CD8+ T-cells were more frequently observed in patients with invalid results than in patients with valid results. CD4+ T-cell depletion in the T-SPOT.TB® mimic experiment reduced the population of IFN-γ producing CD8+ T cells.

**Conclusion:**

T-SPOT.TB® may be invalidated by the interaction between Tax-expressing CD4+ T-cells and cytotoxic CD8+ T-cells. Moreover, HTLV-1-associated immune reactions due to contact between these cells may be unlikely to occur in QFT using whole blood. Therefore, our results reveal the superiority of QFT over T-SPOT.TB® as a screening test for LTBI in HTLV-1-positive patients with RD.

## 1 Introduction

IFN-γ release assays (IGRAs) are a screening test for latent tuberculosis infection (LTBI) that measures IFN-γ production against tuberculosis antigens in T-cells. Two types of IGRAs, including T-SPOT.TB^®^ and QuantiFERON-TB (QFT), are recommended as screening tests for LTBI in patients with rheumatoid arthritis (RA) and connective tissue disease who require immunosuppressive treatment, including biologics (1).

Human T-cell leukemia virus type 1 (HTLV-1) is a retrovirus that causes adult T-cell leukemia-lymphoma (ATL) and HTLV-1-associated myelopathy/tropical spastic paraparesis (HAM/TSP) (2). Japan is one of the leading endemic regions for HTLV-1 infections globally, with approximately 658,000 HTLV-1 carriers (3) and an estimated 4,000 new infections annually (4). HTLV-1 carriers have had weakened acquired immunity to recall antigens and are more susceptible to infectious diseases, such as tuberculosis and strongyloides (5–8). Based on these previous reports, some immunological abnormalities are induced mainly in lymphocytes infected with HTLV-1, and the host’s immune system may be modified, but the detailed mechanism remains unclear.

The LTBI screening test, T-SPOT.TB^®^, appears to be invalid in HTLV-1 carriers and patients with HTLV-1-positive RA because of the increasing number of negative control spots (9, 10). The number of negative control spots increased because peripheral blood (PB) mononuclear cells (PBMCs) from patients with HTLV-1-positive RA produce IFN-γ even in the absence of tuberculosis antigen stimulation (10). Screening tests for LTBI have been recommended in the practice of rheumatic diseases (RDs), but the following research questions remain unanswered: “Does T-SPOT.TB^®^ tend to be invalid in HTLV-1-positive RD as well as in HTLV-1-positive RA?” “Is QFT more reliable as a screening test for LTBI compared to T-SPOT.TB^®^?” “Does HTLV-1 infection affect the QFT results?”.

Therefore, this study investigated the assessment results of T-SPOT.TB^®^ in HTLV-1-positive RD. Additionally, we verified the use of QFT for patients with HTLV-1-positive RD who could not be determined as LTBI by T-SPOT.TB^®^. Moreover, we performed a detailed molecular biological analysis of IFN-γ autonomously producing cells regarding the increase in negative control spots, which determine T-SPOT.TB^®^ inability.

## 2 Materials and methods

### 2.1 Study design and participants

The HTLV-1 RDs Miyazaki Registry was conducted in September 2017 at the Miyazaki University Hospital and Zen-jin-kai Hospital in Miyazaki Prefecture, Japan. This registry aimed to clarify the impact of HTLV-1 infection on the clinical features of patients with RD and to investigate whether immunosuppressive therapies modify the risk factors associated with the development of ATL in patients with HTLV-1-positive RDs. All participants were diagnosed with RDs based on the diagnostic criteria for each disease and screened for HTLV-1 infection. Accordingly, this registry enrolled 47 patients with HTLV-1-positive RD. These patients were expected to periodically visit the hospitals for clinical assessment and sample collection.

The median duration between the proviral load (PVL) measurement and the T-SPOT.TB^®^ test, including the *in vitro* study, is also provided. Blood samples for HTLV-1 PVL measurement, the T-SPOT.TB^®^ assay and *in vitro* experiments, were obtained during periodic hospital visits. The median interval between PVL measurement and T-SPOT.TB^®^ performance for all participants was 9.5 days, with the valid and invalid groups having 12- and 6-day intervals, respectively. The median interval between PVL measurement and *in vitro* experiments was 1.5 days. It has been suggested that HTLV-1 PVL values may not be significantly affected even by several years of immunosuppressive therapy (11). Therefore, we believe that the short intervals in this study did not significantly affect our results.

The participants of the present study were selected from this registry. The inclusion criterion of this study is as follows: patients with HTLV-1-positive RD who underwent T-SPOT.TB^®^ assay (Oxford Immunotec, Oxford, UK) in this registry. The reason for performing T-SPOT.TB^®^ assay was to detect LTBI before initiating immunosuppressive therapy. All clinical information evaluated during LTBI screening, such as a T-SPOT.TB® assay results, HTLV-1 infection status, and immunosuppressive therapy regimen, were collected from the medical records of these participants.

### 2.2 Measurement of IGRAs

The SRL Clinical Laboratory Company (Tokyo, Japan) performed T-SPOT.TB^®^ assays (Oxford Immunotec), following the manufacturer’s instructions (12). Briefly, the assays were considered invalid if the negative control spot count was >10 (high negative control) or if the positive control spot count was <20 (low positive control).

QFT Gold Plus^®^ (QIAGEN, Hilden, Germany) was investigated at BML, Inc. (Tokyo, Japan), following the manufacturer’s instructions (13). QFT was judged to be positive when TB1,2 (IU/mL) was ≥0.35 and Nil (IU/mL) was ≥25% and to be negative when TB1,2 (IU/mL) was <0.35 or Nil (IU/mL) was <25%. However, when the negative control Nil value (IU/mL) is >8.0 (high negative control) or the positive control mitogen value (IU/mL) is <0.5 (low positive control), the determination is invalid.

### 2.3 Measurement of HTLV-1 PVL

PB samples were obtained from patients with HTLV-1-positive RD during follow-up clinical visits. The HTLV-1 PVL was measured using PBMCs separated from PB by Ficoll-based density gradient centrifugation. DNA was purified from PBMCs with the QIAamp Blood DNA Midi Kit (Qiagen) and concentrated to 0.5 μg/μL via ethanol precipitation. Real-time PCR was performed using a LightCycler 2.0 thermal cycler (Roche Diagnostics, Mannheim, Germany) to measure the HTLV-1 pX region and the human albumin gene (14). HTLV-1 PVL values in PBMCs were measured in duplicate, and the number of copies per 100 PBMCs was calculated. These HTLV-1 PVL values were stored in the Miyazaki HTLV-1 RDs Registry database.

### 2.4 HTLV-1-infected cell analysis system flow cytometry (HAS-flow) analysis

We evaluated HTLV-1-infected cells by flow cytometry (FCM) during routine blood sampling for research purposes. Whole blood samples in EDTA tubes were obtained from the participants, and HAS-Flow was performed to detect the expression levels of CADM1 and CD7 in CD4+ T lymphocytes, following previously reported methods (15). As described previously (16), the ratio of CADM1+ CD7dim and CADM1+ CD7neg was defined as the D region (D) and N region (N), respectively, and the CADM1+ CD4+ cell population was defined as the D+N region (D+N). A FACSCalibur FCM (BD Biosciences) was used to analyze stained cells with FlowJo X software (Tree Star, San Diego, CA, USA) for data analysis. The results of HAS-Flow have been stored in the Miyazaki HTLV-1 RDs Registry database and were used for analysis.

### 2.5 Cell isolation for culture experiments

Heparinized whole PB samples for culture experiments were obtained from patients during follow-up clinical visits. PBMCs were separated from heparinized whole PB samples by Ficoll-based density gradient centrifugation. Whole PB samples were obtained from five patients with valid T-SPOT.TB^®^ results and five with invalid T-SPOT.TB^®^ results, all enrolled in the HTLV-1 RD Miyazaki Registry Study.

### 2.6 Magnetic cell sorting

CD4+, CD8+, or Tax-tetramer+ T-cells were depleted from whole PBMCs using magnetic cell separation. Uncultured PBMCs were labeled with either mouse anti-human CD4-FITC-conjugated mAb (OKT4; Biolegend, San Diego, CA, USA), mouse anti-human CD8-FITC-conjugated mAb (HIT8a; Biolegend), or phycoerythrin (PE)-labeled HLA-A*24:02 Tax301-309 tetramer (MBL Tokyo, Japan). Anti-FITC microbeads (Miltenyi Biotec, Bergisch Gladbach, Germany) or anti-PE microbeads (Miltenyi Biotec) were then added and incubated for 15 min at 4°C. The antibody and microbead-labeled PBMCs were magnetically sorted at room temperature (20°C–25°C) using MS columns inserted into a MACS separator (Miltenyi Biotec). We analyzed cell population frequencies in sorted and unsorted samples using a FACSCalibur FCM (BD Biosciences) after staining for the surface expression of mouse anti-human CD3-PE-conjugated mAb (UCTH1; Biolegend), mouse anti-human CD4-PE/Cyanine7-conjugated mAb (SK3; Biolegend), and mouse anti-human CD8-allophycocyanin (APC)-conjugated mAb (RPA-T8; Biolegend) to test the purity of CD4+ and CD8+ T-cell depletion.

### 2.7 Analysis of IFN-γ-producing cells

Whole PBMCs or CD4+ T-cell-depleted PBMCs were suspended in AIM V medium (Thermo Fisher Scientific, Waltham, MA, USA) at a concentration of 2.5 × 10^5^ cells/100 μL. Then, 100 μL of this cell suspension was seeded into a 96-well, round-bottom plate and cultured at 37°C with 5.0% CO_2_ for 16–24 h. Brefeldin A (20 μg/mL) (Sigma, St. Louis, MO, USA) was then added to inhibit protein transport to the Golgi apparatus, and the PBMCs were incubated for an additional 4 h. We performed cell membrane permeabilization using IntraPrep (BD Biosciences, San Diego, CA) to detect intracytoplasmic antigens. PBMCs were incubated with mAbs for 20 min at room temperature for cell surface staining. The antibodies used were mouse anti-human CD3-PE-conjugated mAb (UCTH1; Biolegend), mouse anti-human CD4-PE/Cyanine7-conjugated mAb (SK3; Biolegend), mouse anti-human CD8-APC-conjugated mAb (RPA-T8; Biolegend), mouse anti-human IFN-γ-FITC-conjugated mAb (RUO; BD Biosciences), mouse IgG1-PE/Cyanine7-conjugated mAb (MOPC-21; Biolegend), mouse IgG1-APC-conjugated mAb (MOPC-21; Biolegend), and mouse IgG2b-FITC-conjugated mAb (RUO; BD Biosciences). FACSCalibur FCM (BD Biosciences) was used to analyze stained cells. FlowJo X software (Tree Star, San Diego, CA, USA) was used for the analysis.

### 2.8 Analysis of Tax-expressing cells

Whole PBMCs or CD8+ T-cell-depleted PBMCs were suspended in AIM V medium (Thermo Fisher Scientific) at a concentration of 2.5 × 10^5^ cells/100 μL. Then, 100 μL of this cell suspension was seeded into a 96-well, round-bottom plate and cultured at 37°C with 5.0% CO_2_ for 16–24 h. Following culture, a human FoxP3 staining kit (eBioscience, San Diego, CA, USA) was used for nuclear staining to detect the viral antigen Tax, according to the manufacturer’s protocol. The antibodies used included mouse anti-human CD3-PE-conjugated mAb (UCTH1; Biolegend), mouse anti-human CD4-PE/Cyanine7-conjugated mAb (SK3; Biolegend), mouse anti-human CD8-APC-conjugated mAb (RPA-T8; Biolegend), mouse IgG1-PE/Cyanine7-conjugated mAb (MOPC-21; Biolegend), mouse IgG1-APC-conjugated mAb (MOPC-21; Biolegend), mouse IgG2b-FITC-conjugated mAb (RUO; BD Biosciences), and FITC-conjugated mAb against Tax (LT-4; kindly provided by Tanaka Y, Ryukyu University) (17). Stained cells were analyzed using a FACSCalibur FCM (BD Biosciences). We used the FlowJo X software (Tree Star, San Diego, CA, USA) for data analysis.

### 2.9 Analysis of Tax-specific cytotoxic T lymphocytes

This assay used uncultured PBMCs derived from patients. We stained the PBMCs with PE-conjugated HLA-A*02:01 Tax11-19 tetramer (MBL) and HLA-A*24:02 Tax301-309 tetramer (MBL) along with fluorescence-conjugated mAbs to analyze Tax11-19 or Tax301-309- cytotoxic T lymphocytes (CTLs), following the manufacturer’s instructions. The mAbs used included mouse anti-human CD4-PE/Cyanine7-conjugated mAb (SK3; Biolegend), mouse anti-human CD8-APC-conjugated mAb (RPA-T8; Biolegend), PE-conjugated HLA-A*02:01 control tetramer (MBL), and HLA-A*24:02 control tetramer (MBL). We used PE-conjugated HLA-A*02:01 Tax11-19 tetramer (MBL) and HLA-A*24:02 Tax301-309 tetramer (MBL); thus, we included patients with positive HLA-A*02:01 and HLA-A*24:02 in our analysis. To confirm HLA-A*02:01 or HLA-A*24:02 positivity, PBMCs were stained with anti-HLA-A*02:01-APC-conjugated mAb (RUO; Biolegend) and anti-HLA-A*24:02-PE-conjugated mAb (17A10; MBL), following the manufacturer’s instructions. All 10 participants in this study demonstrated at least one HLA-A*02:01 or HLA-A*24:02 allele.

### 2.10 IFN-γ enzyme-linked immunospot (ELISPOT) assay

A human IFN-γ ELISPOT Kit (Mabtech) was used for the IFN-γ ELISPOT assay, following the manufacturer’s instructions. Whole and depleted (CD4, CD8, and Tax-tetramer) PBMCs obtained from patients with invalid T-SPOT.TB^®^ results were used in this assay. The cells were seeded into 96-well ELISPOT plates at 2.5 × 10^5^ cells per well and incubated for 24 h at 37°C in a 5% CO_2_ incubator. PBMCs stimulated with phorbol myristate acetate (PMA, 50 ng/mL; Sigma) and ionomycin (2 μM; Nakarai) were used as positive controls, while PBMCs treated with brefeldin A (5 μg/mL; Biolegend) to suppress IFN-γ release were used as negative controls. Spontaneous IFN-γ production was detected by using the human IFN-γ ELISPOT Kit (Mabtech) and the ELIPHOTO Counter (Minerva Tech), and all experiments were performed in triplicate.

### 2.11 Statistical analysis

GraphPad Prism version 9.3.1 was used for statistical analyses. Median with interquartile for continuous variables and count with proportion for categorical data were used to describe the participants’ characteristics. Group comparison on age, PVL, corticosteroid dosage, and spot count between T-SPOT.TB^®^ valid and invalid groups was conducted using Mann–Whitney *U* test. The sex ratio, prevalence ratio of HTLV-1-associated diseases, prevalence ratio of underlying RDs, corticosteroid usage ratio, immunosuppressant or immunomodulatory usage ratio, and biologics agent or JAK inhibitors usage ratio between the two groups were compared using Fisher’s exact test. Group comparison on the percentage of cells, such as IFN-γ+ CD8+ T cells in CD3+ T cells, Tax-tetramer+ T cells in CD8+ T cells, Tax+ T cells in CD4+ T cells, etc., between the two groups was conducted using Mann–Whitney *U* test in FCM analysis. Spearman’s rank correlation coefficient was used to analyze the correlations between the spot count in the negative control of T-SPOT.TB^®^ and PVL, the spot count in the negative control of T-SPOT.TB^®^ and the IFN-γ levels in the Nil value of QFT, and the spot count in the negative control of T-SPOT.TB^®^ and the proportion of HTLV-1-infected cells in the HAS-Flow assay used. The cutoff value for PVL with high specificity and sensitivity to differentiate between valid and invalid T-SPOT.TB^®^ was evaluated using receiver operating characteristic (ROC) curve. Youden’s index was calculated for all points of the ROC curves, and the maximum index value was used to select the optimal cutoff value for PVL. *P*-values <0.05 were considered significant.

### 2.12 Study approval

The Ethics Committee of the University of Miyazaki Hospital (approval no. O-0236) and Zenjinkai Hospital approved this study protocol. This study adhered to the Ethical Guidelines for Medical and Health Research Involving Human Subjects and the Declaration of Helsinki. All patients provided written informed consent.

## 3 Results

### 3.1 Characteristics of valid and invalid T-SPOT.TB^®^ in patients

**Figure 1:**
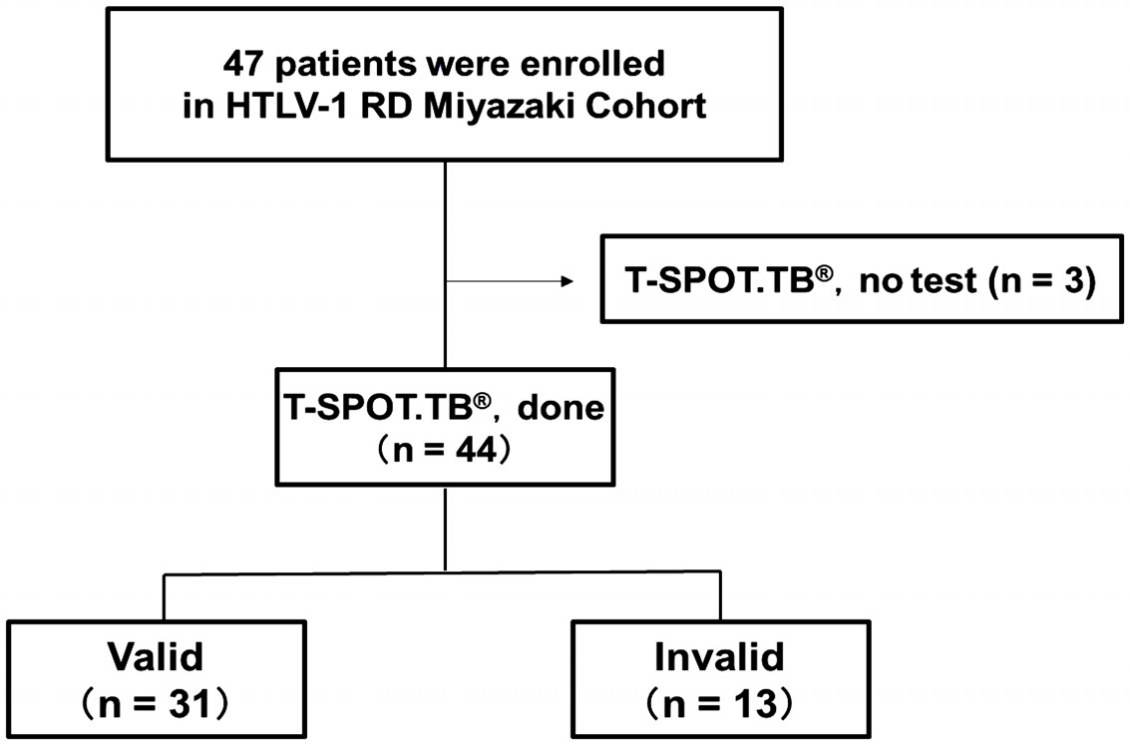
Number of participants with rheumatic disease (RD) who underwent T-SPOT.TB^®^ in HTLV-1 RD Miyazaki Registry. This registry enrolled 47 patients. Of the 47 patients, 44 underwent T-SPOT.TB^®^ assay during their clinical course. These 44 patients were categorized into two groups according to the T-SPOT.TB^®^ assay results as valid and invalid groups.

This study included 44 patients with RD who underwent T-SPOT.TB^®^ examination from the enrolled 47 patients in the HTLV-1 RD Miyazaki Registry Study from September 2017 to December 2022 (**Figure 1**). These patients were categorized into valid and invalid groups based on the T-SPOT.TB^®^ results in the medical record. **Table 1** shows the clinical characteristics during T-SPOT.TB^®^.

**Table 1:** Characteristics of T-SPOT.TB^®^ valid and invalid patients.

	T-SPOT.TB^®^		*P*-value
	Valid (*n* = 31)	Invalid (*n* = 13)	
Age, years (IQR)	67 (63–76.5)	69 (63–75)	0.867^a^
Female, *n* (%)	23 (74.2)	10 (76.9)	1.000^b^
HTLV-1 infection status, *n* (%)			
AC	30 (96.8)	9 (69.2)	0.022^b^
ATL	0	2 (15.4)	0.083^b^
HAM/TSP	1 (3.2)	2 (15.4)	0.204^b^
HU	0	1 (7.7)^c^	0.295^b^
PVL (copies/100 PBMCs) (IQR)	0.49 (0.13–1.68)	2.45 (1.39–6.61)	0.002^a^
Underlying rheumatic diseases, *n* (%)			
RA	17 (54.8)	6 (46.2)	0.744^b^
SjS	8 (25.8) ^d^	5 (38.5)^e^	0.478^b^
SSc	2 (6.5)	3 (23.1)	0.144^b^
PM/DM	4 (12.9)	1 (7.7)	1.000^b^
SLE	1 (3.2)	1 (7.7)	0.509^b^
GPA	0	1 (7.7)	0.296
IgG4-RD	1 (3.2)	0	1.000^b^
MPA	1 (3.2)	0	1.000^b^
PN	1 (3.2)	0	1.000^b^
PMR	1 (3.2)	0	1.000^b^
RS3PE	1 (3.2)	0	1.000^b^
Patients treated with corticosteroids, *n* (%)	24 (77.4)	7 (53.8)	0.155^b^
Corticosteroid dosage (mg/day) (IQR)	7.5 (4.8–10.5)	5 (4.5–15)	0.757^a^
Patients treated with immunosuppressant or immunomodulator, *n* (%)	20 (64.5)	5 (38.5)	0.182^b^
Patients treated with biologic agents or JAK inhibitor, *n* (%)	4 (12.9)	2 (15.4)	1.000^b^
Spot count in T-SPOT.TB^®^ assay panels			
Negative control (IQR)	1 (0–3)	133 (58–220)	<0.001^a^
Positive control (IQR)	234 (93.5–353.5)	537 (299–558)	0.005^a^

Values are expressed as medians interquartile range (IQR). Percentages (%) are calculated based on the total number of patients in each group unless indicated otherwise.

AC, asymptomatic carrier; ATL, adult T-cell leukemia lymphoma; HAM/TSP, HTLV-1-associated myelopathy/tropic spastic paraparesis; HU, HTLV-1-associated uveitis; PVL, proviral load; PBMCs, peripheral blood mononuclear cells; RA, rheumatoid arthritis; SjS, Sjögren’s syndrome; SSc, systemic scleroderma; PM/DM, polymyositis/dermatomyositis; SLE, systemic lupus erythematosus; GPA, granulomatosis polyangiitis; IgG4-RD, IgG4-related disease; MPA, microscopic polyangiitis; PN, polyarteritis nodosa; PMR, polymyalgia rheumatica; RS3PE, remitting seronegative symmetrical synovitis with pitting edema syndrome.

a Mann–Whitney *U* test was performed to compare statistical differences between the groups.

b Fisher’s exact test was performed to compare statistical differences between the groups.

c One case overlapped HAM/TSP and HU.

d Four cases overlapped RA and SjS, one case overlapped SSc and SjS, and one case overlapped DM and SjS.

e Two cases overlapped RA and SjS, one case overlapped SSc and SjS, and one case overlapped SLE and SjS.

Of the 44 patients with HTLV-1-positive RD, 13 (29.5%) demonstrated invalid T-SPOT.TB^®^ results. No differences in age, sex, underlying RD, and treatment regimen were found between the two groups. PVL was higher in the invalid group than the valid group (*P* = 0.002). The cutoff value for PVL to differentiate between the valid and invalid results was 1.11 copies/100 PBMCs using the ROC curve analysis. Therefore, patients with PVL values exceeding 1.11 copies/100 PBMCs are likelier to present with an invalid T-SPOT.TB^®^ result (area under the curve = 0.791, sensitivity = 0.92, and specificity = 0.64) (**Supplementary Figure S1**). The comparison of spot counts of negative controls in T-SPOT.TB^®^ and PVL in 43 patients with RD revealed a correlation between these values (*r* = 0.52, *P* < 0.001) (**Supplementary Figure S2**). We excluded one patient with ATL because of an extremely high PVL value compared with other patients in this study. Ultimately, the correlation analysis was performed on 43 patients. Approximately 30% of the patients in the invalid group had concomitant HTLV-1-associated diseases, such as ATL and HAM/TSP. The spot counts of both the negative and positive controls in T-SPOT.TB^®^ were higher in the invalid group than in the valid group (133 vs. 1, *P* < 0.001, 537 vs. 234, *P* = 0.005, respectively).

### 3.2 QFT is a potentially useful IGRA in T-SPOT.TB^®^-invalid HTLV-1-positive RD

QFT testing was performed on 20 and 13 patients of the valid and invalid groups, respectively. **Table 2** presents the T-SPOT.TB^®^ and QFT results in these 33 patients. The QFT results were determinable in all patients. This study indicated one T-SPOT.TB^®^-invalid case who was diagnosed as LTBI due to a positive QFT result. The comparison of spot counts of negative controls in T-SPOT.TB^®^ and the Nil values in QFT indicated a correlation between these values (*r* = 0.57, *P* < 0.01) (**Supplementary Figure S3**). This correlation analysis excluded one patient with high negative control spot counts in T-SPOT.TB^®^ (747) and Nil values in QFT (7.98).

**Table 2:** Comparison of T-SPOT.TB^®^ and QFT results in patients with HTLV-1-positive rheumatic disease.

Case	HTLV-1 infection status	Rheumatic disease	Spot count in T-SPOT.TB^®^ assay panels					IFN-γ levels in QFT assay tubes (IU/mL)				
			ESAT-6	CFP10	Negative control	Positive control	Result	TB1 value	TB2 value	Nil value	Mitogen value	Result
1	AC	SSc	483	484	747	550	Invalid	0.56	<0.05	7.98	3.78	Negative
2	AC	RA	479	606	661	695	Invalid	0.07	<0.05	0.15	>10.00	Negative
3	HAM/TSP	RA	55	47	244	590	Invalid	0.15	0.21	0.33	>10.00	Negative
4	AC	RA	148	170	220	318	Invalid	<0.05	<0.05	0.09	9.61	Negative
5	AC	DM	174	149	209	591	Invalid	<0.05	<0.05	0.06	>10.00	Negative
6	ATL	SLE, SjS	105	80	181	558	Invalid	0.51	0.21	1.98	7.82	Positive
7	HAM/TSP, HU	RA, SjS	69	115	133	263	Invalid	<0.05	<0.05	<0.05	9.21	Negative
8	ATL	SSc, SjS	81	90	81	382	Invalid	0.15	0.06	0.41	>10.00	Negative
9	AC	RA	48	44	73	208	Invalid	<0.05	<0.05	0.11	>10.00	Negative
10	AC	RA, SjS	50	45	58	537	Invalid	<0.05	<0.05	0.09	5.88	Negative
11	AC	SSc	34	21	53	556	Invalid	0.13	0.15	0.26	>10.00	Negative
12	AC	GPA	12	20	33	299	Invalid	<0.05	<0.05	<0.05	4.25	Negative
13	AC	SjS	0	0	18	284	Invalid	<0.05	<0.05	<0.05	>10.00	Negative
14	HAM/TSP	RA	6	3	10	35	Negative	<0.05	0.14	0.2	>10.00	Negative
15	AC	RS3PE	11	8	10	77	Negative	<0.05	<0.05	<0.05	>10.00	Negative
16	AC	SSc	6	5	6	297	Negative	<0.05	<0.05	0.05	>10.00	Negative
17	AC	RA, SjS	2	0	4	334	Negative	<0.05	<0.05	0.06	>10.00	Negative
18	AC	RA	2	7	3	286	Negative	<0.05	<0.05	0.12	>10.00	Negative
19	AC	SjS	2	2	2	854	Negative	<0.05	<0.05	0.07	>10.00	Negative
20	AC	RA	0	0	1	234	Negative	<0.05	<0.05	<0.05	>10.00	Negative
21	AC	DM	4	2	0	79	Negative	<0.05	<0.05	<0.05	0.67	Negative
22	AC	RA	0	0	0	103	Negative	<0.05	<0.05	0.09	>10.00	Negative
23	AC	DM	0	0	0	91	Negative	<0.05	<0.05	<0.05	>10.00	Negative
24	AC	RA	0	0	0	122	Negative	<0.05	<0.05	<0.05	>10.00	Negative
25	AC	RA	0	0	0	378	Negative	<0.05	<0.05	<0.05	6.76	Negative
26	AC	RA	0	2	0	113	Negative	<0.05	<0.05	0.18	>10.00	Negative
27	AC	RA	0	0	0	408	Negative	<0.05	<0.05	0.08	>10.00	Negative
28	AC	RA, SjS	0	0	0	305	Negative	<0.05	<0.05	<0.05	>10.00	Negative
29	AC	RA	0	0	0	504	Negative	<0.05	<0.05	0.06	>10.00	Negative
30	AC	RA, SjS	0	0	0	71	Negative	<0.05	<0.05	<0.05	>10.00	Negative
31	AC	RA	0	0	0	230	Negative	<0.05	<0.05	<0.05	6.66	Negative
32	AC	RA, SjS	0	0	0	66	Negative	<0.05	<0.05	0.05	0.62	Negative
33	AC	SLE	0	0	0	155	Negative	<0.05	<0.05	<0.05	2.18	Negative

ESAT-6, early secretary antigen target 6; CFP10, culture filtrate protein 10; AC, asymptomatic carrier; ATL, adult T-cell leukemia lymphoma; HAM/TSP, HTLV-1-associated myelopathy/tropic spastic paraparesis; HU, HTLV-1 associated uveitis; RA, rheumatoid arthritis; DM, dermatomyositis; SLE, systemic lupus erythematosus; SjS, Sjögren’s syndrome; SSc, systemic scleroderma; GPA, granulomatosis polyangiitis; RS3PE, remitting seronegative symmetrical synovitis with pitting edema syndrome.

### 3.3 HTLV-1-infected cell population among CD4+ T cells tends to be higher in patients with invalid T-SPOT.TB^®^ compared to those with valid results

**Figure 2:**
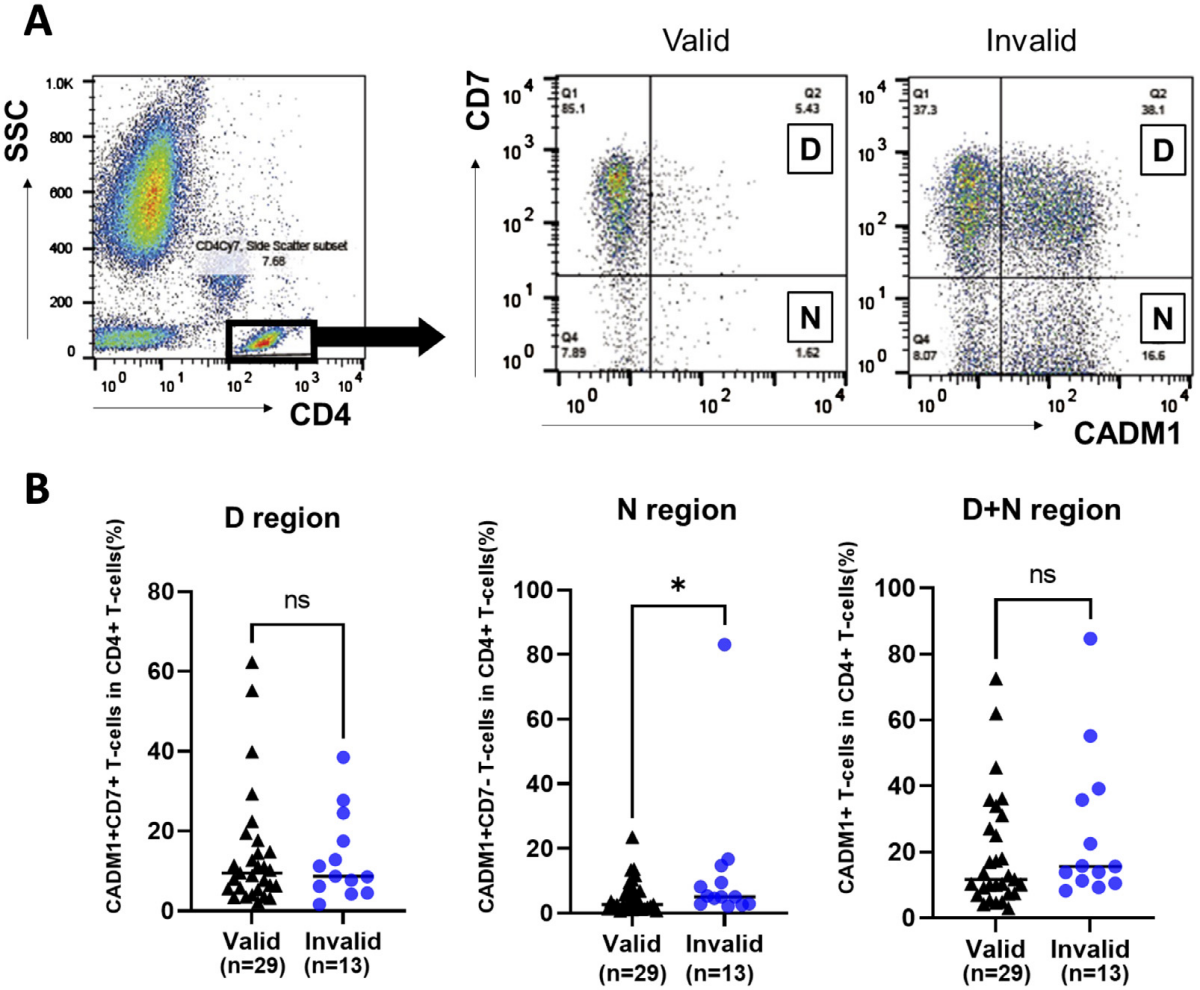
Comparison of the population of HTLV-1-infected cells in participants with rheumatic disease (RD) who demonstrated valid or invalid T-SPOT.TB^®^ assay results using HAS-Flow. The population of HTLV-1-infected cells was determined using HAS-Flow. **(A)** Representative data of CADM1 *versus* CD7 plot in CD4+ T-cells in participants with RD who exhibited valid or invalid T-SPOT.TB^®^ assay results. Gating of the CD4+ T-cell population in peripheral blood. CADM1 *versus* CD7 plot of CD4+ T-cell population. The upper-right and lower-right regions were named D and N, respectively, following the previous study. **(B)** The population of HTLV-1-infected cells in D, N, and D+N regions was compared between the valid (*n* = 29) and invalid (*n* = 13) groups. The bold horizontal line indicates the median values. *P < 0.05 by Mann–Whitney *U* test.

The HTLV-1-infected cell population among CD4+ T cells in the PB of patients with valid and invalid T-SPOT.TB^®^ result was evaluated using HAS-Flow (**Figure 2A**). The proportion of HTLV-1-infected cell populations, such as CADM1+ CD7dim+ T-cells and CADM1+ CD7-T-cells within the CD4+ T-cell subset, was higher in patients with invalid T-SPOT.TB^®^ results compared to those with valid results. The median percentage of CADM1+ CD7dim+ CD4+ T cells (D) was 9.49% in the valid group (*n* = 29) and 8.73% in the invalid group (*n* = 13) (**Figure 2B**). Conversely, the median percentage of CADM1+ CD7-CD4+ T cells (N) was significantly higher in the T-SPOT.TB^®^ invalid group compared to the valid group (5.04 vs. 2.66, *P* = 0.043). No significant difference was found in the median percentage of CADM1+ CD4+ T-cells (D+N) between the two groups (**Figure 2B**, **Supplementary Table S1**). A correlation was observed between the negative controls’ spot counts on T-SPOT.TB^®^ testing and the percentage of N region cells (*r* = 0.40, *P* < 0.05) (**Supplementary Figure S4**). In this correlation analysis, one ATL participant was excluded because of high CADM1+ CD7- T cells compared with other participants in this study. Therefore, this correlation analysis was performed on 41 participants.

### 3.4 CD8+ T cells autonomously producing IFN-γ exist in PBMCs of patients with invalid T-SPOT.TB^®^

**Figure 3:**
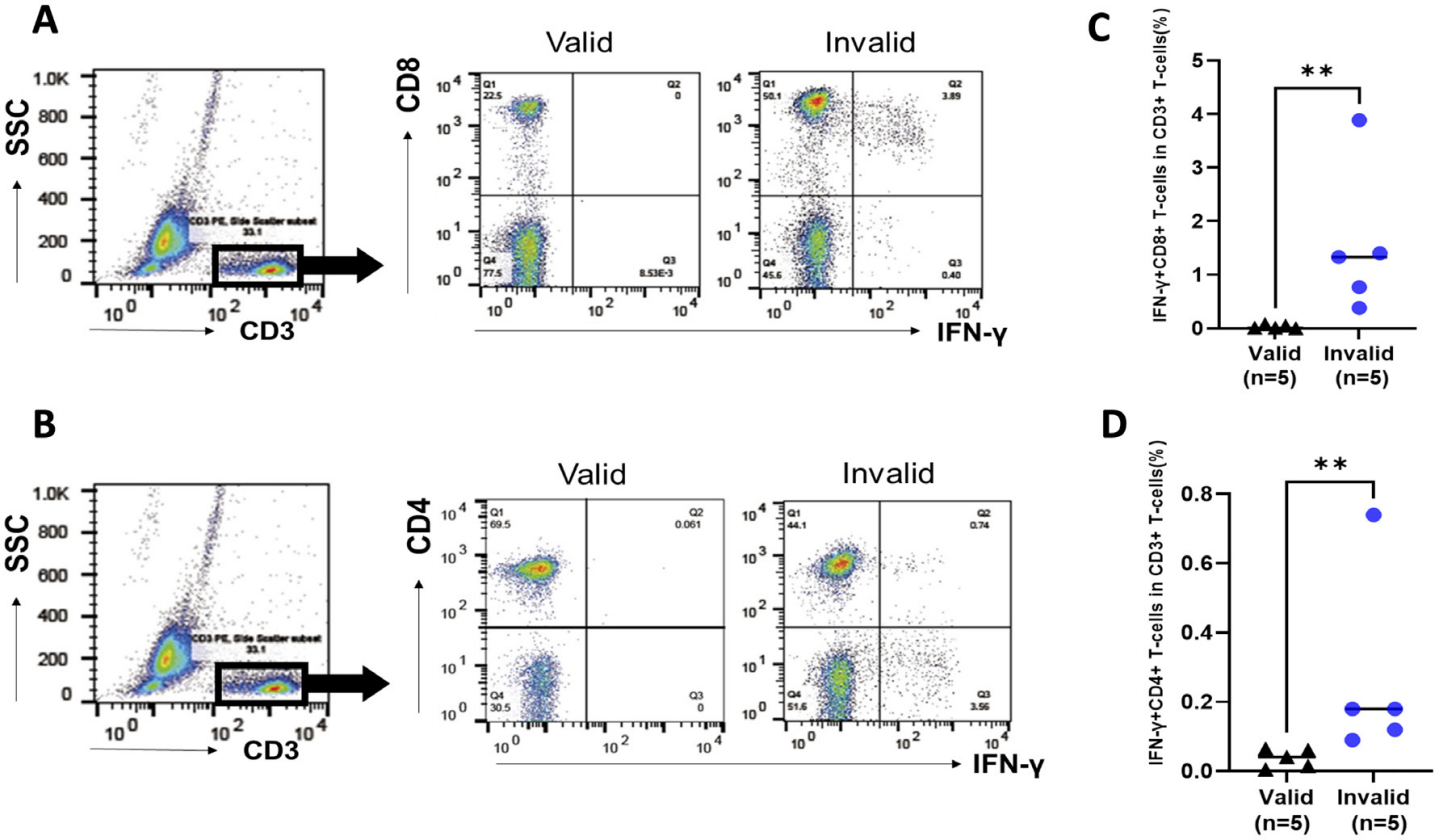
Analysis of IFN-γ-producing cells in peripheral blood mononuclear cells (PBMCs) in participants with rheumatic disease (RD) who demonstrated valid and invalid T-SPOT.TB^®^ results. PBMCs were obtained from participants with RD cultured without stimulation. PBMCs were collected after 24 h, and the population of IFN-γ-producing cells was analyzed using flow cytometry. **(A)** Representative data of CD8 *versus* IFN-γ plot of CD3+ T-cell population in participants with RD who exhibited valid or invalid T-SPOT.TB^®^ assay results. **(B)** Representative data of CD4 *versus* IFN-γ plot of CD3+ T-cell population in participants with RD who demonstrated valid or invalid T-SPOT.TB^®^ assay results. **(C)** Population of IFN-γ-producing CD8+ T cells and **(D)** IFN-γ-producing CD4+ T cells in participants with RD who showed valid (*n* = 5) or invalid (*n* = 5) T-SPOT.TB^®^ assay results. The bold horizontal line indicates the median values. **P < 0.01 by Mann–Whitney *U* test.

PBMCs that were obtained from patients with valid and invalid T-SPOT.TB^®^ were cultured without stimulations, mimicking T-SPOT.TB^®^’s cell culture condition to identify IFN-γ-autonomously producing cells. PBMCs were obtained from five patients with valid and five with invalid T-SPOT.TB^®^ having HTLV-1-positive RD. **Supplementary Table S2** shows the clinical characteristics of the 10 patients with HTLV-1-positive RD. **Figure 3** illustrates the representative data of FCM analysis of IFN-γ producing cells. Both IFN-γ producing CD8+ T cells and CD4+ T cells were observed in PBMCs obtained from the invalid (but not from the valid) group (**Figures 3A, B**). One patient in the valid group detected no IFN-γ producing cells; the median percentages of the IFN-γ+ CD8+ T-cell population were higher in invalid patients than in those valid (1.33% vs. 0.01%, *P* < 0.01) (**Figure 3C**). The median percentages of the IFN-γ+ CD4+ T-cells population were also higher in the invalid group (0.18% vs. 0.04%, *P* < 0.01) (**Figure 3D**). There were no differences between the proportion of CD8+ and CD4+ T cells among the CD3+ T-cell population in patients with invalid T-SPOT.TB^®^ (**Supplementary Figure S5A**), and the proportion of IFN-γ autonomously producing T cells in the CD8+ T-cell population was higher than those in the CD4+ T-cell population (*P* < 0.05) (**Supplementary Figure S5B**).

### 3.5 Tax-specific CTLs produce IFN-γ in response to Tax-expressed HTLV-1-infected cells

**Figure 4:**
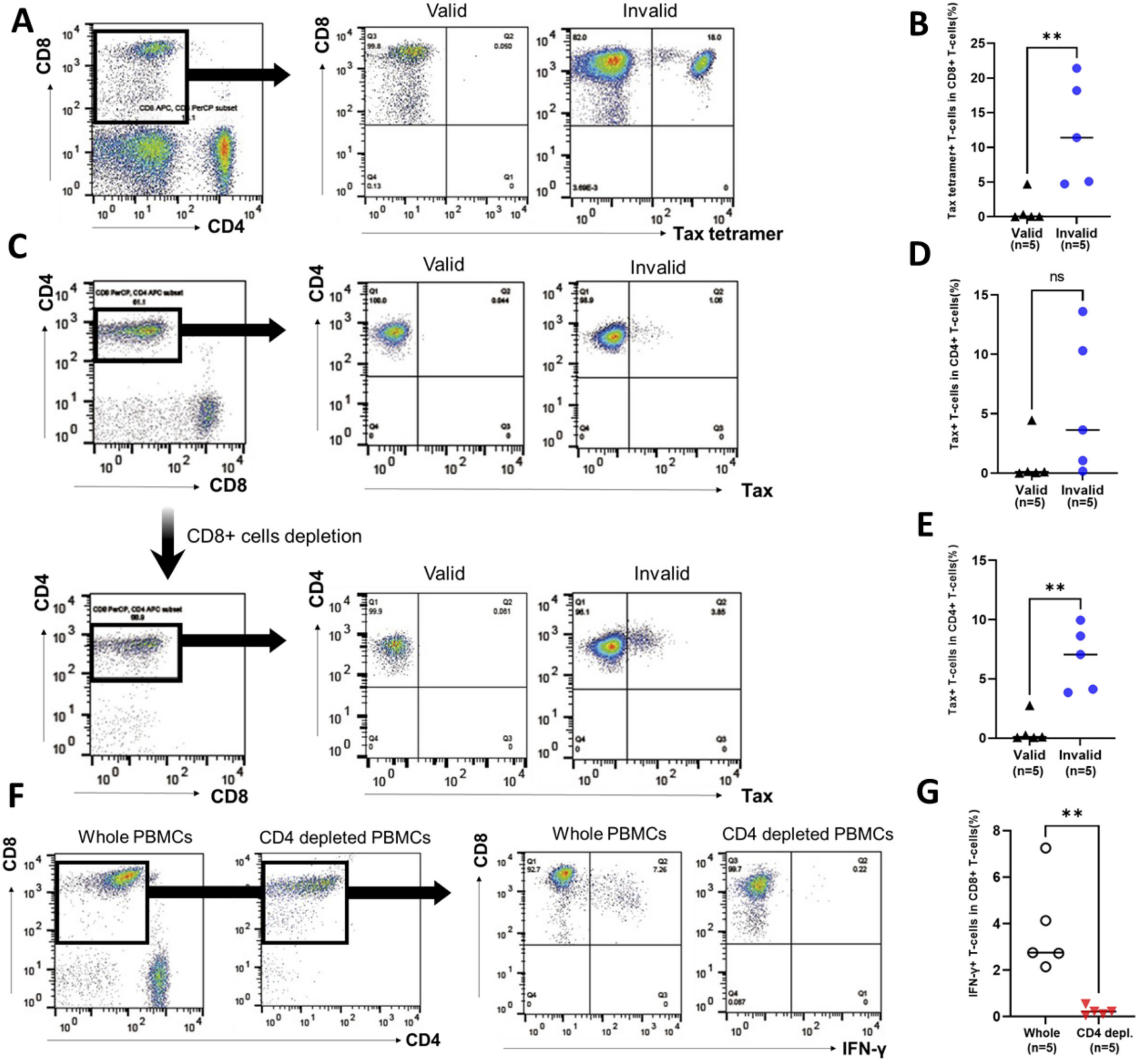
Population of Tax-specific cytotoxic lymphocytes (CTLs) and Tax-expressed HTLV-1-infected cells in participants with rheumatic diseases (RD) who demonstrated valid or invalid T-SPOT.TB^®^ assay results. Peripheral blood mononuclear cells (PBMCs) were obtained from participants with RD, and the population of Tax-specific CTLs was determined using flow cytometry. **(A)** Representative data of CD8 *versus* Tax-tetramer plot in participants with RD who exhibited valid or invalid T-SPOT.TB^®^ assay results. **(B)** Population of Tax-specific CTLs in participants with RD who showed valid (*n* = 5) or invalid (*n* = 5) T-SPOT.TB^®^ assay results. PBMCs were obtained from participants with RD cultured without stimulation. PBMCs were collected after 24 h, and the population of Tax-expressed CD4+ T cells or IFN-γ-producing CD8+ T cells was determined using flow cytometry. **(C)** Representative data of CD4 *versus* Tax plot in whole PBMCs and CD8+ T-cell-depleted PBMCs including Tax-specific CTLs of RD participants who demonstrated valid or invalid T-SPOT.TB^®^ assay results. **(D)** Population of Tax-expressed CD4+ T cells in whole PBMCs in participants with RD who showed valid (*n* = 5) or invalid (*n* = 5) T-SPOT.TB^®^ assay results. **(E)** Population of Tax-expressed CD4+ T cells in PBMC-depleted CD8+ T cells, including Tax-specific CTLs, in participants with RD who showed valid (*n* = 5) or invalid (*n* = 5) T-SPOT.TB^®^ assay results. **(F)** Representative data of CD8 *versus* IFN-γ plot of CD3+ T-cell population plot in whole PBMCs and CD4+ T-cell-depleted PBMCs in participants with RD who demonstrated invalid T-SPOT.TB^®^ assay results. **(G)** IFN-γ producing CD8+ T cells in whole PBMCs and CD4+ T-cell-depleted PBMCs in participants with RD who exhibited invalid (*n* = 5) T-SPOT.TB^®^ assay results. The bold horizontal line indicates the median values. **P < 0.01 by Mann–Whitney *U* test.

The Tax-tetramer+ CD8+ T-cell population was analyzed as Tax-specific CTLs in uncultured PBMCs. **Figure 4A** shows the representative data of Tax-tetramer *versus* CD8 plot in PBMCs of patients with RD who demonstrated valid or invalid T-SPOT.TB^®^ assay results. Tax-specific CTL population was observed in PBMCs obtained from a patient with RD who exhibited invalid T-SPOT.TB^®^ assay results. Conversely, only a small number of Tax-specific CTL populations were observed in PBMCs obtained from a patient with RD who demonstrated valid T-SPOT.TB^®^ assay results. The median percentages of the Tax-specific CTL population in PBMCs were higher in patients with RD with invalid T-SPOT.TB^®^ assay results than in those with valid T-SPOT.TB^®^ assay results (11.4 vs. 0.05, *P* < 0.01) (**Figure 4B**). A part of IFN-γ-producing Tax-specific CTLs exists in the CD8+ T-cell population (**Supplementary Figures S6A, B**).

The expression of Tax in the CD4+ T-cell population was analyzed in whole PBMCs and CD8+ T-cell-depleted PBMCs, considering CD4+ T cells as HTLV-1-infected cells. PBMCs were cultured by mimicking the cell culture condition of T-SPOT.TB^®^ negative control. **Figure 4C** shows the representative data of Tax *versus* CD4 plot in whole PBMCs and CD8+ T-cell-depleted PBMCs of patients with RD who demonstrated valid or invalid T-SPOT.TB^®^ assay results. Tax-expressed CD4+ T-cell population was observed in whole PBMCs and CD8+ T-cell-depleted PBMCs obtained from a patient with RD who exhibited invalid T-SPOT.TB^®^ assay results. Conversely, only a small number of Tax-expressed CD4+ T-cell populations were observed in PBMCs obtained from a patient with RD who demonstrated valid T-SPOT.TB^®^ assay results. There was a high percentage of Tax-expressed CD4+ T-cell population in the whole PBMCs of patients with RD with invalid T-SPOT.TB^®^ assay results compared to those with valid T-SPOT.TB^®^ assay results, but not significant (**Figure 4D**). Conversely, the median percentages of Tax-expressed CD4+ T-cell population in CD8+ T-cell-depleted PBMCs of patients with RD with invalid T-SPOT.TB^®^ assay results were higher than those with valid T-SPOT.TB^®^ assay results (7.06 vs. 0.11, *P* < 0.01) (**Figure 4E**). The increase of Tax-expressing cells after CD8+ T-cell depletion was observed in three of five patients with invalid T-SPOT.TB^®^ assay results. The population of tax-specific CTLs was larger in these three patients than in the other two (**Table 3**).

**Table 3:** Population of Tax-specific cytotoxic T cells, Tax-expressed HTLV-1-infected cells, and IFN-γ production CD8+ T cells in RD participants who showed invalid T-SPOT.TB^®^ assay results.

Case	PVL (copies/100 PBMCs)	Tax tetramer+ T cells in CD8+ T cells (%)	Tax+ T cells in CD4+ T cells (%)			IFN-γ+ T cells in CD8+ T-cells (%)		
			Whole cells	CD8-depleted PBMCs	Fold change	Whole cells	CD4-depleted PBMCs	Fold change
RD2	9.01	21.4	1.06	3.85	3.63	4.14	0.22	0.05
RD1	19.1	18.2	3.63	7.06	1.94	7.26	0.22	0.03
RD4	3.59	11.4	0.16	4.13	25.8	2.76	0.11	0.04
RD5	6.14	5.10	10.3	9.96	0.97	2.73	0.54	0.20
RD3	8.32	4.73	13.6	8.62	0.63	2.15	0.06	0.03

RD, rheumatic disease; PVL, proviral load; PBMCs, peripheral blood mononuclear cells.

The population of IFN-γ-producing CD8+ T-cells was analyzed in whole PBMCs and CD4+ T-cell-depleted PBMCs. PBMCs that were obtained from patients with RD who demonstrated invalid T-SPOT.TB^®^ assay results were cultured for 24 h without stimulation, mimicking the cell culture condition of T-SPOT.TB^®^ negative control. **Figure 4F** shows the representative data of IFN-γ *versus* CD8 plot in whole PBMCs and CD4+ T-cell-depleted PBMCs. An IFN-γ-producing CD8+ T-cell population was observed in whole PBMCs, but not in CD4+ T-cell-depleted PBMCs. The median percentages of the IFN-γ+ CD8+ T-cells population were higher in whole PBMCs than in CD4+ T-cell-depleted PBMCs (2.76 vs. 0.22, *P* < 0.01) (**Figure 4G**). **Table 3** shows the results of IFN-γ production in whole PBMCs and CD4+ T-cell-depleted PBMCs of five patients with invalid T-SPOT.TB^®^. Among all patients, the IFN-γ+CD8+ T-cell population decreased due to CD4+ T-cell depletion from PBMCs. The percentage of IFN-γ-producing CD8+ T cells in whole PBMCs was the highest at 7.26% in the RD1 case, but it was significantly decreased in CD4+ T-cell-depleted PBMCs to 0.22%. The fold change was 0.03.

### 3.6 Tax-specific CTLs are the main IFN-γ-producing cells in the ELISPOT assay mimicking T-SPOT.TB^®^

**Figure 5:**
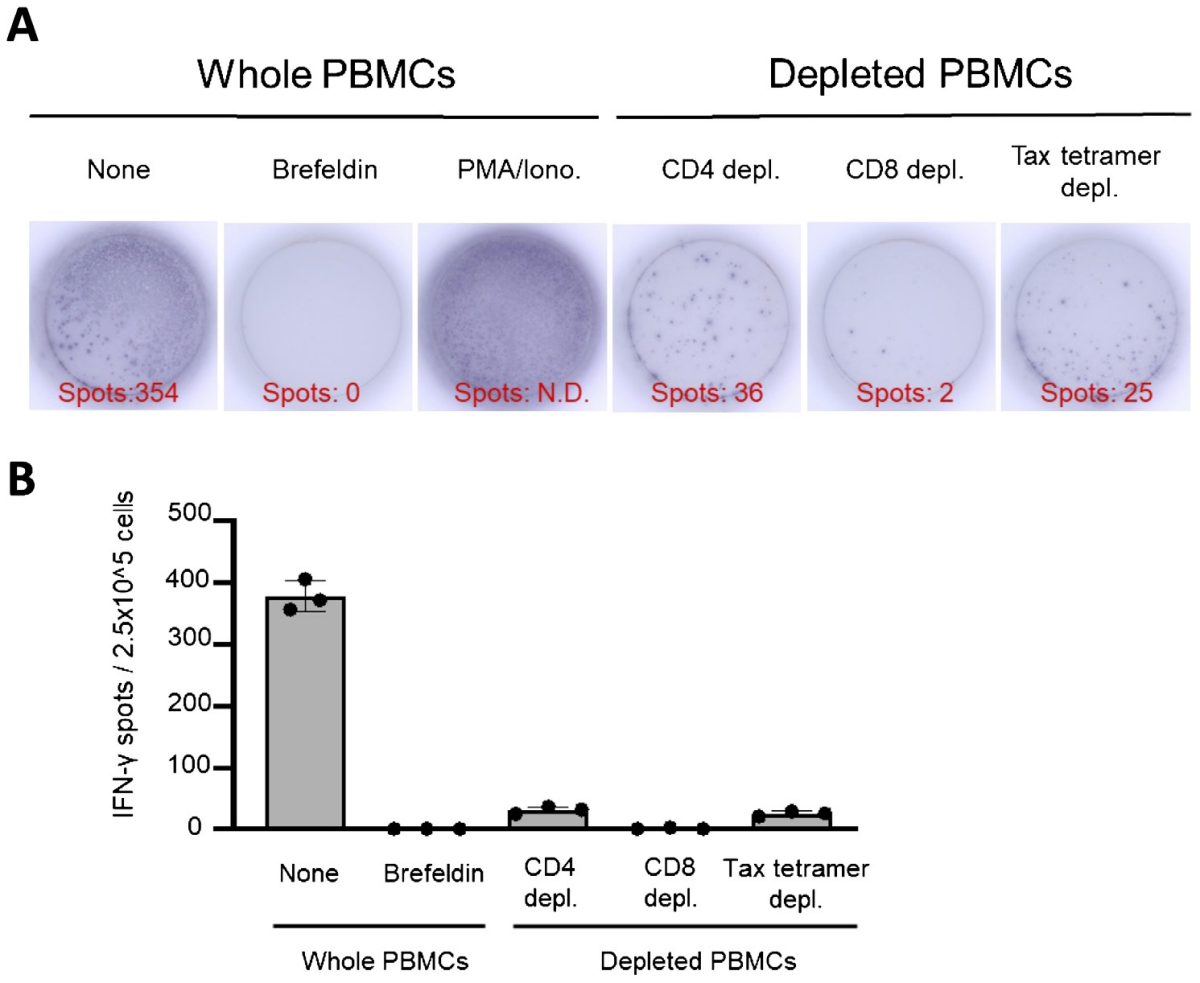
IFN-γ-ELISPOT assay mimicking control panels of T-SPOT.TB^®^ after depletion of CD4, CD8, and Tax-specific cytotoxic lymphocytes (CTLs). An ELISPOT assay mimicking the control panels of T-SPOT.TB^®^ was performed using whole peripheral blood mononuclear cells (PBMCs) or CD4, CD8, and Tax-specific CTL-depleted PBMCs. **(A)** Representative data of the IFN-γ-ELISPOT assay. After 24 h of culture, 354 spots were observed in culture conditions without stimulation. No spots were observed in the brefeldin A treatment condition. Numerous spots were observed in phorbol myristate acetate (PMA)/ionomycin (Iono) treatment conditions as a positive control. Decreased spots were confirmed in each culture condition of CD4, CD8, and Tax-specific CTL-depleted PBMCs, respectively. **(B)** Number of IFN-γ spots in each culture condition. The IFN-γ ELISPOT assay was repeated thrice. The bar presents mean ± SD.

An ELISPOT assay mimicking the control panels of T-SPOT.TB^®^ was performed using whole PBMCs, CD4+ T cells, CD8+ T cells, and Tax-specific CTL-depleted PBMCs (**Figure 5**). The ELISPOT assay mimicking the negative control panel of T-SPOT.TB^®^ using whole PBMCs demonstrates 354 spots. None of the spots were observed in the ELISPOT assay using brefeldin-treated whole PBMCs. These results indicate that brefeldin autonomously suppressed IFN release from PBMCs. Additionally, decreased spots were observed in the ELISPOT assay using CD4+ T cells, CD8+ T cells, and Tax-specific CTL-depleted PBMCs (**Figure 5A**). This experiment was conducted thrice independently. Several spots were detected in the ELISPOT assay using whole PBMCs. Conversely, a decrease of these spots was observed in this assay using CD4+ T cells, CD8+ T cells, and Tax-specific CTL-depleted PBMCs (**Figure 5B**).

## 4 Discussion

This study revealed that patients with HTLV-1-positive RD demonstrating invalid results in T-SPOT.TB^®^ exhibited higher HTLV-1 PVL and more HTLV-1-associated diseases. This is the first study to show that QFT may be useful as a screening test for LTBI in patients with HTLV-1-positive RD with invalid T-SPOT.TB^®^ results. The frequency of invalid T-SPOT.TB^®^ results was approximately 0.6%, and it has been a screening test with excellent sensitivity and specificity for LTBI (18). The previous study investigated the usefulness of T-SPOT.TB^®^ as a screening test for LTBI in 311 patients with RD, demonstrating as follows: 85.9% were negative, 14.1% were positive, and no patients were invalid (19). The present study revealed 13 (29.5%) patients with HTLV-1-positive with RD to have invalid results, which is a much higher number than in previous reports. Behcet’s disease in patients and the temperature at sample transfer have been factors causing invalid T-SPOT.TB^®^ results (20, 21). However, this study did not include any patients with Behcet’s disease. The temperature during sample transfer was well controlled, and these factors were considered to not cause invalid T-SPOT.TB^®^. T-SPOT.TB^®^ was invalid in approximately 55% of patients with HTLV-1-positive RA (10). This study revealed that T-SPOT.TB^®^ is also invalid in patients with HTLV-1-positive RD, including RA. However, the patients who participated in this study had only a limited number of underlying RD diseases, and verification in a larger number of patients with a variety of underlying RD diseases was deemed necessary.

The judgment criteria of invalid QFT include a high Nil value of the negative control. This study revealed valid QFT in all patients with HTLV-1-positive RD. A significant positive correlation between the number of negative controls in T-SPOT.TB^®^ and Nil value IFN-γ concentration in QFT was found, but no patient demonstrated a high Nil value of >8 IU/mL which is defined as an invalid result. Excessive IFN-γ production in the negative control of QFT was not observed compared to that in T-SPOT.TB^®^. QFT is an IGRA that uses whole blood samples, whereas T-SPOT.TB^®^ involves culturing PBMCs in 96-well plates. The IFN-γ measurement methods also differ between ELISPOT and enzyme-linked immunosorbent assay. These differences in measurement techniques may contribute to variations in IFN-γ production observed in negative controls. Previous reports have revealed that QFT in patients with RA and RD under immunosuppressive treatment is frequently invalid compared to T-SPOT.TB^®^ because the mitogen value, which serves as a positive control, is low (22, 23). These studies indicated the influence of immunosuppressants on PBMCs, but the positive control demonstrated adequate responses in all patients with RD enrolled in the present study. One patient with invalid T-SPOT.TB^®^ result was QFT-positive and was diagnosed with LTBI in this study. Therefore, we indicate that QFT may be more useful than T-SPOT.TB^®^ as a screening test for LTBI in patients with HTLV-1-positive RA and RD. However, since few patients with RD were evaluated with QFT, we could not rule out the possibility that the analysis of more patients with RD would lead to the identification of additional patients with invalid QFT.

In this study, similar to previous reports, T-SPOT.TB^®^ appeared invalid in patients with RD who had high HTLV-1 PVL (10). The cutoff value for PVL to distinguish between valid and invalid results was determined to be 1.11 copies/100 PBMCs using ROC curve analysis. Patients with PVL values higher than 1.11 copies/100 PBMCs may show invalid T-SPOT.TB^®^ results. However, approximately 30% of patients in the T-SPOT.TB^®^ testable group had PVL values of 1.11 copies/100 PBMCs or more. Therefore, further studies with a larger cohort are needed to establish a more useful cutoff value. The present study demonstrated that the populations of HTLV-1-infected cells, such as CADM1+ CD7-CD4+ T cells and CADM1+ CD4+ T cells, were elevated in patients with HTLV-1-positive RD exhibiting invalid T-SPOT.TB^®^ results compared to those with valid results. The HAS-Flow analysis, developed for real-time analysis of HTLV-1-infected cells, including ATL cells, showed that CADM1-positive cells with downregulated CD7 associated with ATL development may act as ATL progenitor cells (24). This study indicated that the increasing number of negative control spots in T-SPOT.TB^®^ correlated with many HTLV-1-infected cells, including ATL progenitor cells. Moreover, T-SPOT.TB^®^ was more likely to be invalid in patients with HTLV-1-associated diseases such as HAM/TSP and ATL. Therefore, many HTLV-1-infected cells, including ATL progenitor or ATL cells, may increase the likelihood of invalid T-SPOT.TB^®^ results in patients with HTLV-1-positive RD.

PBMCs obtained from HTLV-1-infected individuals autonomously produce IFN-γ when cultured without stimulation (25–27). Additionally, autonomous IFN-γ production was observed in the ELISPOT assay; this may serve as an early diagnostic method for HAM/TSP (28). In particular, CD8+ T cells autonomously produced IFN-γ in PBMCs obtained from patients with HAM/TSP (29). The present study revealed that the predominant population producing IFN-γ was CD8+ T cells in cell culture conditions mimicking T-SPOT.TB^®^ negative control. Additionally, a small number of CD4+ T cells producing IFN-γ were observed. HTLV-1 infection can alter the phenotype of CD4+ T cells to Th1-like helper T cells. These Th1-like HTLV-1-infected CD4+ T cells autonomously produce IFN-γ (30). Therefore, CD8+ T cells and Th1-like HTLV-1-infected CD4+ T cells can affect the T-SPOT.TB^®^ results, potentially causing false-positive TB antigen panel results and increased spot counts in the negative control. These results altogether indicated the involvement of these IFN-γ autonomously producing T cells, predominantly CD8+ T cells, in the invalid T-SPOT.TB^®^ results in patients with HTLV-1-positive RD.

This study revealed many Tax-specific CTLs and Tax-expressing CD4+ T cells in patients with invalid T-SPOT.TB^®^ compared to patients with invalid results. CD4+ T-cell depletion, including HTLV-1-infected cells, from whole PBMCs obtained from patients with invalid T-SPOT.TB^®^ decreased the population of IFN-γ-producing CD8+ T cells. These results indicated that CTLs respond to HTLV-1-infected cells and were considered the main cell population of IFN-γ-producing CD8+ T cells. Tax is one of the accessory genes encoded in the pX region, a characteristic of HTLV-1 provirus, and plays an important role in controlling infection, such as infected cell viral replication and proliferation (31). Additionally, Tax is highly immunogenic, and Tax-expressing HTLV-1-infected cells are easily eliminated by the antiviral immune response (32–34). Tax-specific CTLs targeting Tax play a crucial role in suppressing viral proliferation and may inhibit the malignant progression of infected cells (35). Kubota et al. revealed that Tax-specific CTLs produce IFN-γ when PBMCs obtained from patients with HAM/TSP are cultured for >14 h (29, 36). Additionally, HTLV-1-infected CD4+ T cells obtained from HTLV-1 carriers express Tax after 12–24 h of culture (37). Therefore, the antiviral immune response of CTLs producing IFN-γ may increase the spots in the negative control of T-SPOT.TB^®^ assay in patients with HTLV-1-positive RD.

**Figure 6:**
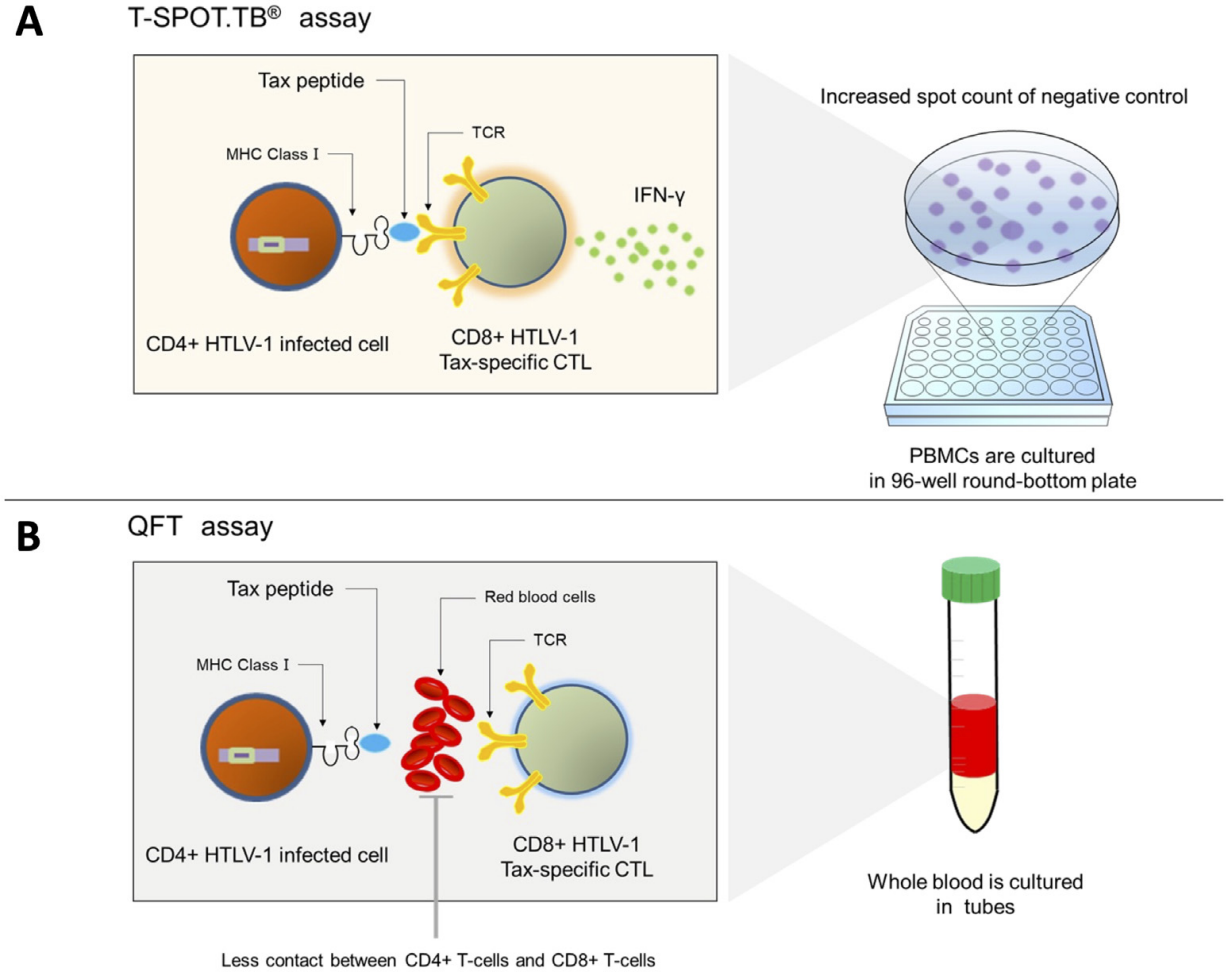
Mechanism of the invalid result of T-SPOT.TB^®^ in patients with positive HTLV-1. **(A)** Tax-specific cytotoxic CD8+ T cells recognize the Tax peptide/MHC-I complex antigen presented on CD4+ T cells and produce IFN-γ in patients with a high HTLV-1 proviral load. Hence, the spot counts of negative control in T-SPOT.TB^®^ may be high. **(B)** Less contact is observed between CD4+-infected cells and CTLs in QFT using whole blood samples and culturing them in tubes, and IFN-γ production may be reduced.

The present study revealed the invalid T-SPOT.TB^®^ results in patients with HTLV-1-positive RD having high HTLV-1 PVL, and the higher the PVL, the higher the number of spot counts in the negative control. The culture conditions of PBMCs in T-SPOT.TB^®^ make contact reactions between Tax-specific CTLs and Tax-expressing infected cells more likely to occur, considering the measurement method of T-SPOT.TB^®^. CTLs produce a large amount of IFN-γ, which increases the number of negative control spots, in PB mononuclear cells, which have a large number of CTLs and HTLV-1-infected cells (**Figure 6A**). Conversely, QFT is an assay using whole blood samples. Red blood cells, white blood cells, and plasma proteins, which are removed in the T-SPOT.TB® assay, may inhibit the contact reaction between HTLV-1-infected cells and CTLs. Therefore, IFN-γ production by CTLs due to an antiviral immune response may be less likely to occur in QFT (**Figure 6B**). However, in a few patients with invalid T-SPOT.TB® results, Tax-expressing CD4+ T cells did not increase after CD8+ T-cell depletion. The ELISPOT assay of this study demonstrated no <10 IFN-γ spots despite Tax-specific CTL depletion. These results indicate the existence of CTLs that react to HTLV-1-related proteins other than Tax. CTLs recognized HTLV-1 basic leucine zipper factor (HBZ), an HTLV-1-related protein as well as Tax, as a viral antigen that causes IFN-γ production from CTLs (38). We could not exclude the possibility that CTLs recognize not only Tax but also HTLV-1-infected cell antigens, such as HBZ, to produce IFN-γ.

This study has several limitations that warrant consideration. First, this study enrolled a small number of patients with HTLV-1-positive RD. Second, only 33 patients with HTLV-1-positive RD were additionally measurable by QFT. Third, the RD of the patients studied were not diverse. Our results demonstrate that QFT may be a useful IGRA in patients with RD who demonstrated invalid T-SPOT.TB^®^ results; verification in a large cohort of patients with RD is necessary to determine whether QFT is useful in patients with RD who exhibited invalid results in T-SPOT.TB^®^. Fourth, based on the culture experiment that mimicked the negative T-SPOT.TB^®^ control, the anti-HTLV-1-Tax immune response of Tax-specific CTL against HTLV-1-infected T cells contributed to the increased spot counts. However, the association between the immune response to other non-Tax HTLV-1-related proteins, such as HBZ, and the invalid T-SPOT.TB^®^ result was unclear.

In summary, patients with HTLV-1-positive RD who demonstrated invalid T-SPOT.TB^®^ results are likely to be HTLV-1 carriers with high PVL. The factor of increasing the number of spots in the negative control of T-SPOT.TB^®^ in patients with HTLV-1-positive RD was considered the induction of IFN-γ production due to the antiviral immune response between Tax-specific CTLs and HTLV-1-infected cells. Similar responses may not be achieved between these cells in QFT compared with T-SPOT.TB^®^, considering the differences of assay methodology. Therefore, our results indicate that QFT may be superior to T-SPOT.TB® as a screening test for LTBI in patients with HTLV-1-positive RD. Many HTLV-1 endemic countries are also endemic areas for tuberculosis. Therefore, QFT may be useful as a screening test for LTBI in people living with HTLV-1 in these areas. In the future, well-powered and highly sensitive studies involving more HTLV-1-infected individuals, with and without RDs, are needed to verify the usefulness of QFT as a screening test for LTBI.

## Data Availability

The original contributions presented in the study are included in the article/**Supplementary Material**. Further inquiries can be directed to the corresponding author.
