# Supplementary material for: Antiviral immune response against HTLV-1 invalidates T-SPOT.TB® results in patients with HTLV-1-positive rheumatic diseases
Source: Front Immunol. 2024 Oct 29;15:1480506. doi: 10.3389/fimmu.2024.1480506 (PMC11554456; doi:10.3389/fimmu.2024.1480506)

Supplementary Material

# Supplemental Figures and Tables

## Supplementary Figures


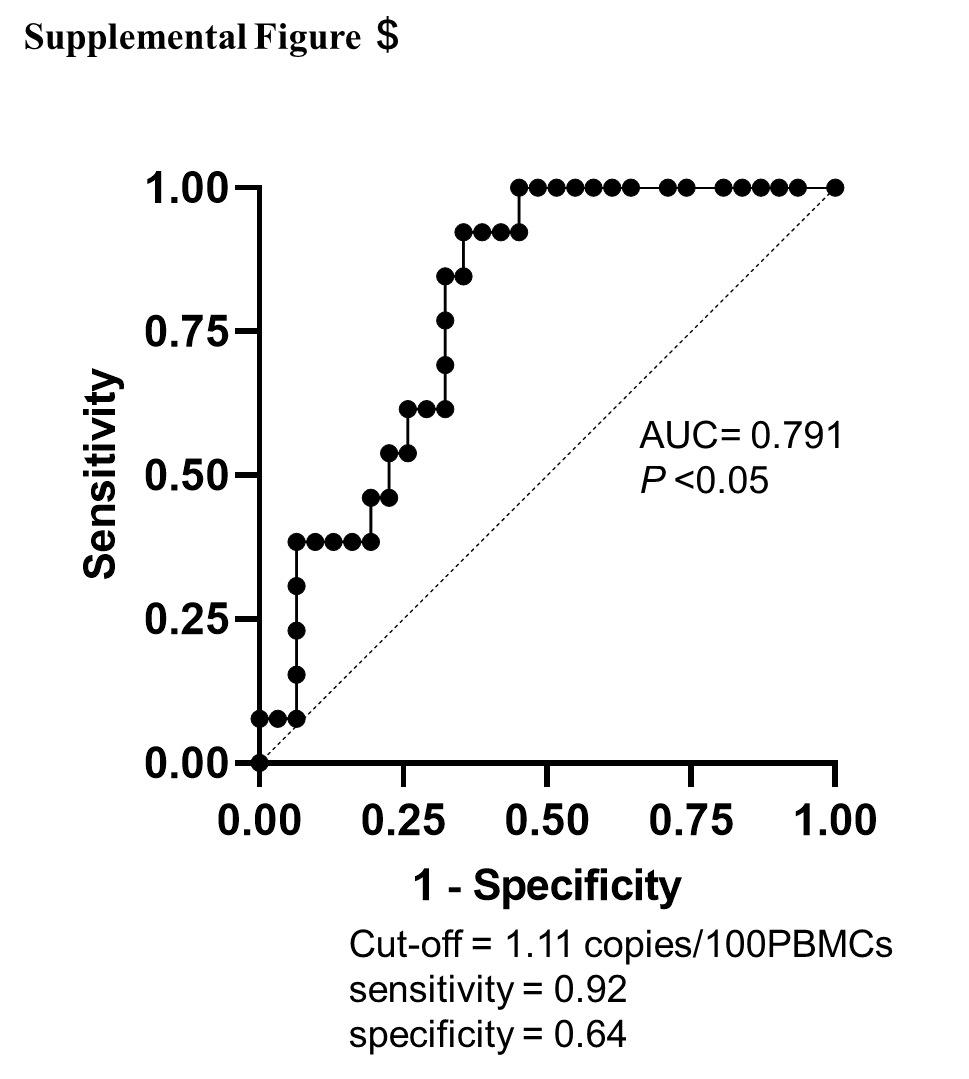


**Supplemental Figure 1. Receiver operating characteristic (ROC) curve analysis of proviral load (PVL) for predicting invalid T-SPOT.TB® results (n = 44).**

This figure shows the ROC analysis of PVL for predicting invalid T-SPOT.TB® results. Using the ROC curve and Youden index for optimal accuracy, the cutoff value of PVL was determined to be 1.11 copies/100 PBMCs. AUC: area under the curve; PBMCs: peripheral blood mononuclear cells.


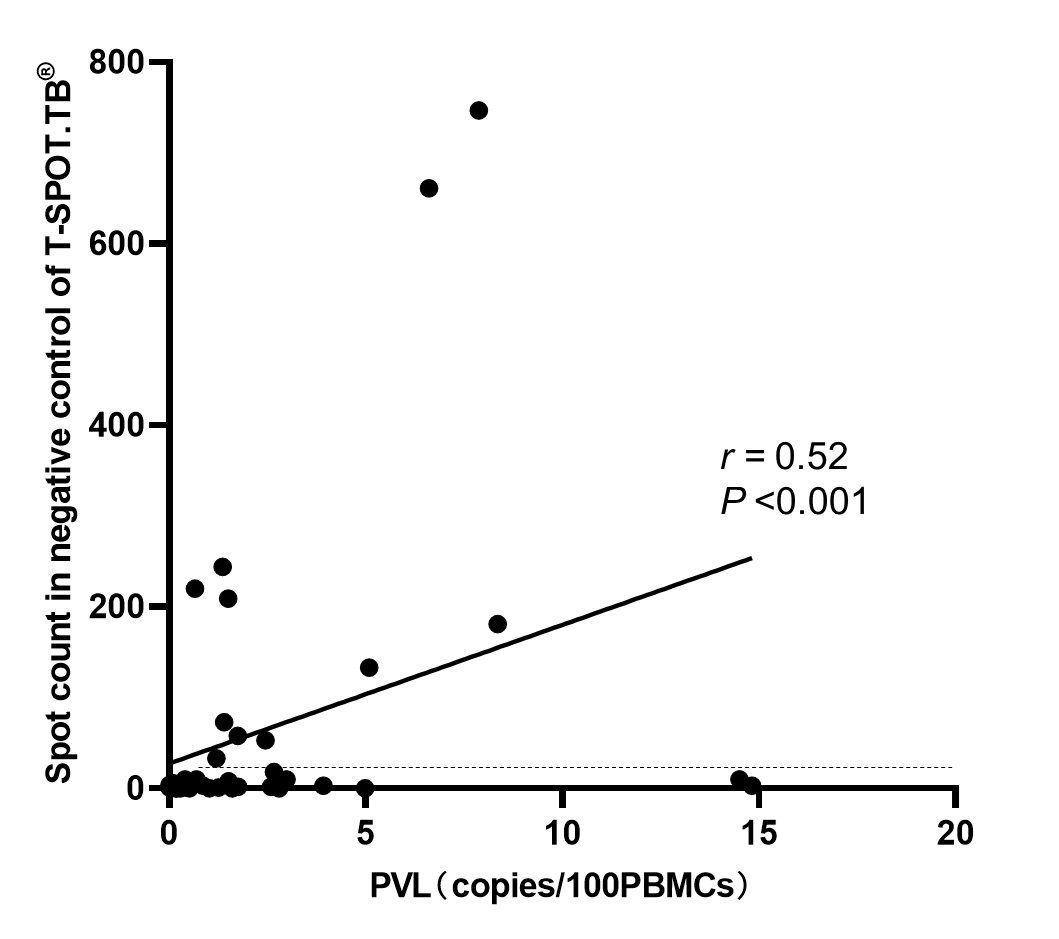


**Supplementary Figure 2.** **Correlation between spot count in the negative control of T-SPOT.TB® assay and proviral load (PVL) (n = 43)**

This scatter plot shows the relationship between the spot count and the negative control of the T-SPOT.TB® assay and PVL (copies/100 PBMCs) in 43 participants with rheumatic disease. We excluded one case of ATL with abnormally high PVL. The dotted line indicates the spot count threshold at which T-SPOT.TB® results are considered invalid.

**
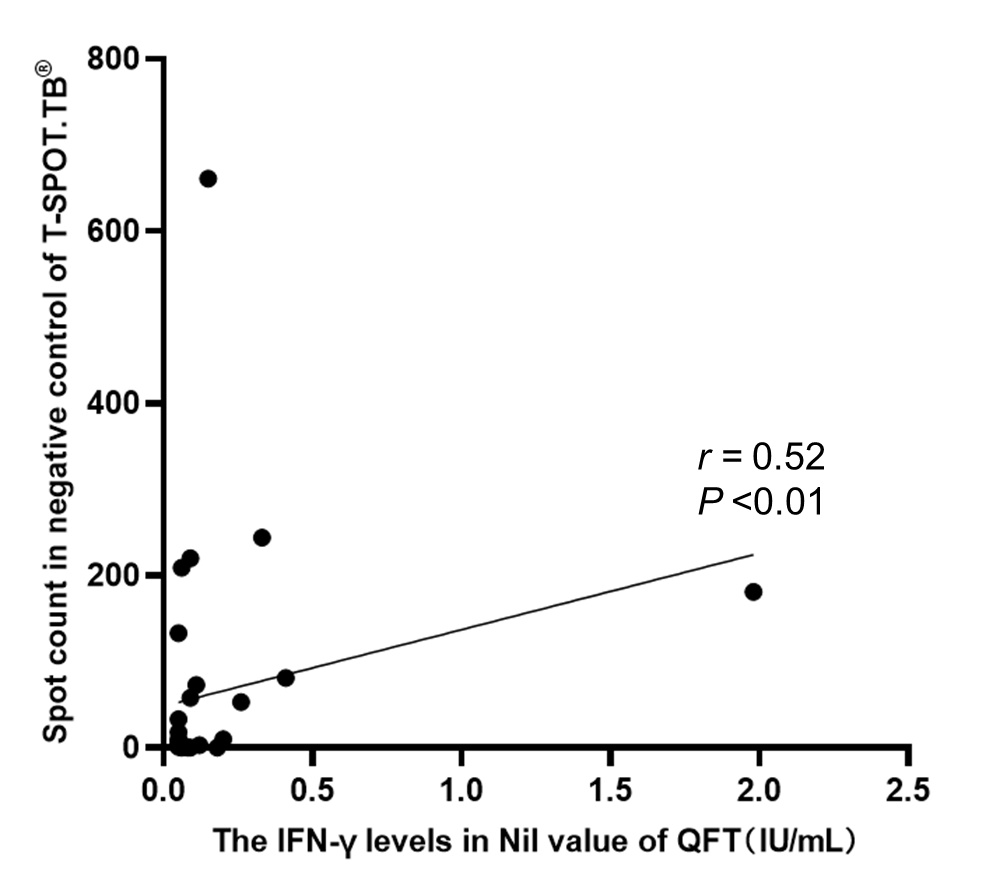
**

**Supplemental Figure 3.** **Correlation between spot count in the negative control of T-SPOT.TB® assay and IFN-γ levels in Nil value of QFT (n = 32)**

This scatter plot shows the relationship between the spot count and the negative control of the T-SPOT.TB® assay and the IFN-γ levels in the Nil value of the QFT assay in 32 participants with rheumatic disease. We excluded one patient carrier with abnormally high IFN-γ levels in the Nil value of the QFT.


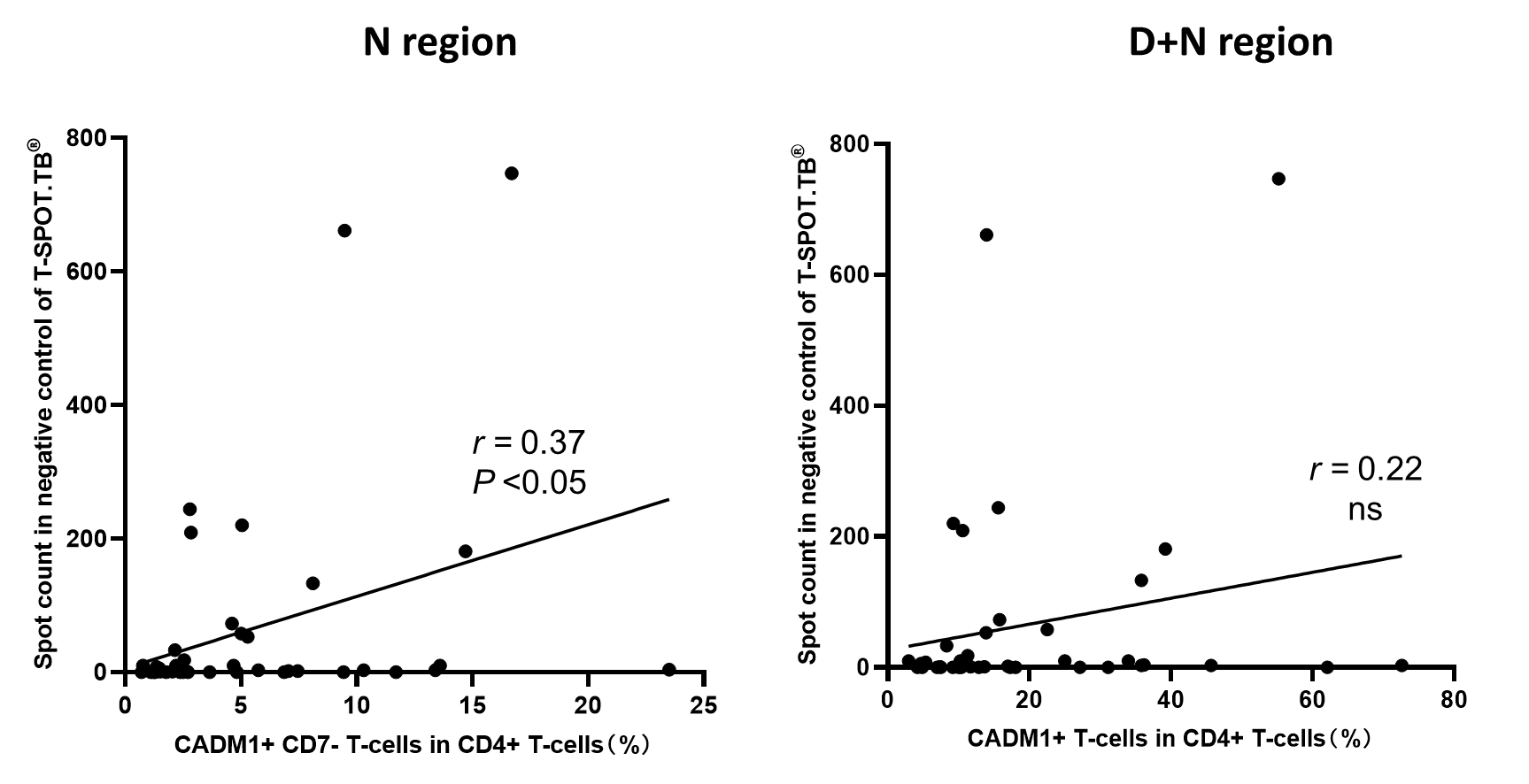


**Supplemental Figure 4. Correlation between spot count in the negative control of T-SPOT.TB® assay and proportion of HTLV-1-infected cells population** **in HTLV-1-infected cell analysis system flow cytometry (HAS-Flow) (n = 41)**

This scatter plot shows the relationship between the spot count in the negative control of the T-SPOT.TB® assay and the proportion of HTLV-1-infected cell population as measured by the HTLV-1-infected cell analysis system flow cytometry (HAS-Flow) in 41 participants with rheumatic disease. We excluded one case of ATL with an abnormally high proportion of CADM1+ CD7- T-cells in CD4+ T-cells.


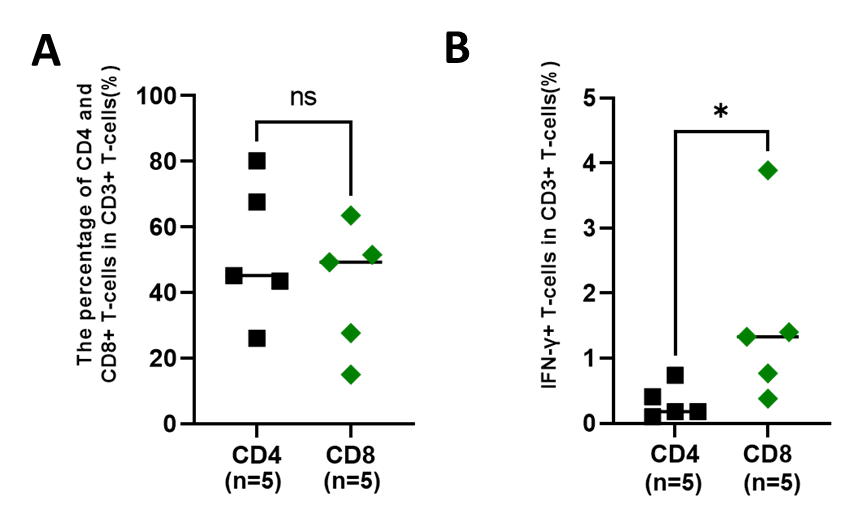


**Supplemental Figure 5. Analysis of IFN-γ-producing cells in patients with invalid T-SPOT.TB® by Flowcytometry**

The percentages of CD4+ T-cells and CD8+ T-cells and the presence of IFN-γ-producing cell population in CD3+ T-cells population in T-SPOT.TB®️ invalid patients (n = 5). **(A)** The median percentages of CD4+ T-cells and CD8+ T-cells in CD3+ T-cells were 45.2% and 49.3%, respectively, with no significant difference. **(B)** The median percentages of the IFN-γ+ T-cell population were 0.18% and 1.33%, respectively, indicating many IFN-γ-producing cells in CD8+ T-cells.

Bold lines indicate the median values. **P* < 0.05, by Mann–Whitney U test.


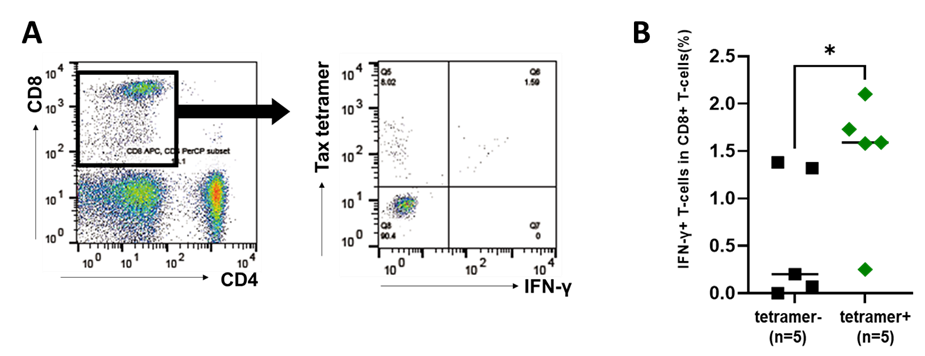


**Supplemental Figure 6. IFN-γ producing cells in Tax-specific cytotoxic T-cells (CTLs)**

Peripheral blood mononuclear cells (PBMCs) were obtained from participants with rheumatic disease (RD), who demonstrated invalid T-SPOT.TB® assay results, cultured without stimulation. PBMCs were collected after 24 h, and the population of IFN-γ producing cells in Tax-specific CTLs was analyzed using flow cytometry. (**A**) The representative data of Tax tetramer versus IFN-γ plot of CD8+T-cell population. (**B**) The population of IFN-γ producing CD8+T-cells in Tax-specific CTLs in participants with RD who exhibited invalid T-SPOT.TB® assay results (n = 5). The population of IFN-γ producing CD8+T-cells is larger in Tax-specific CTLs compared to in Tax-nonspecific CTLs. The bold horizontal line indicates the median values. **P* < 0.05, by Mann–Whitney U test.


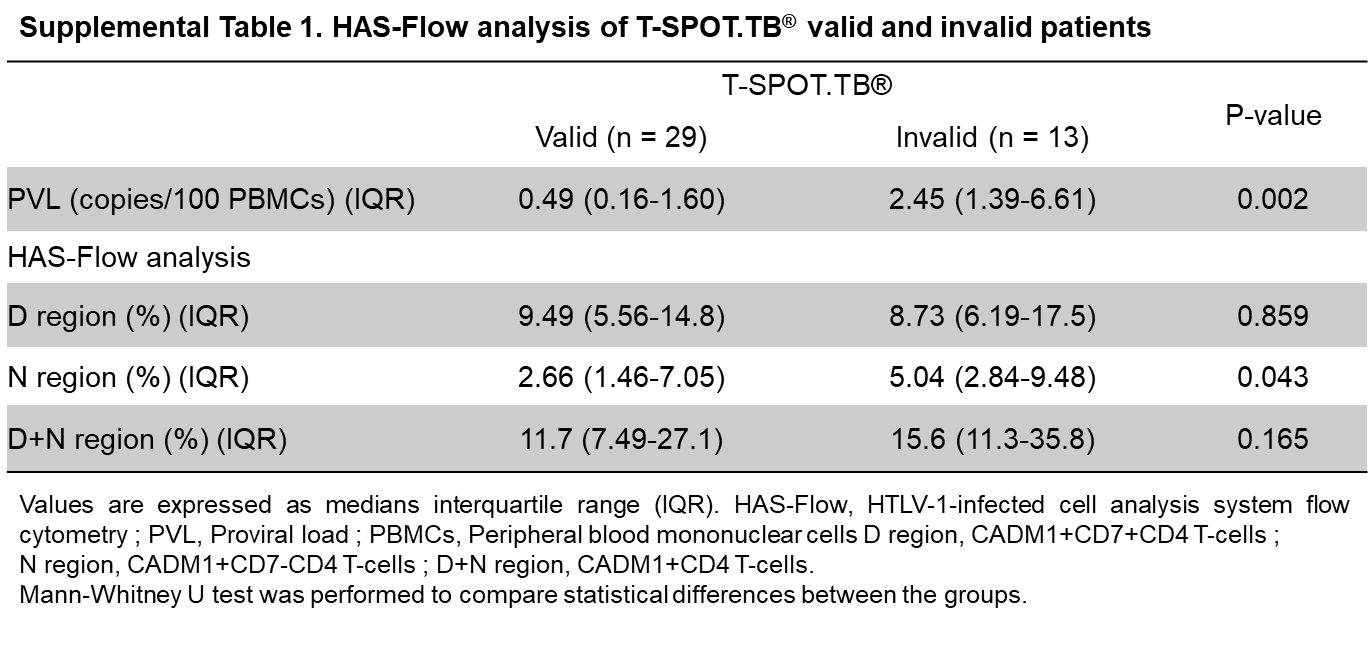


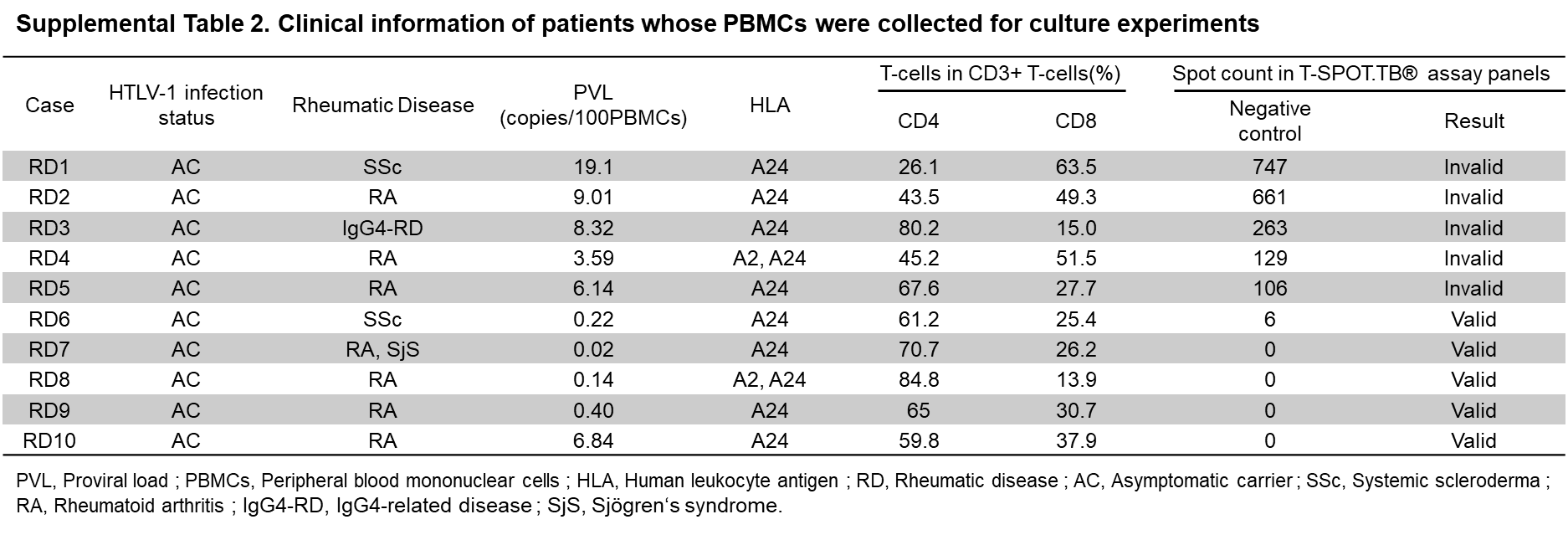


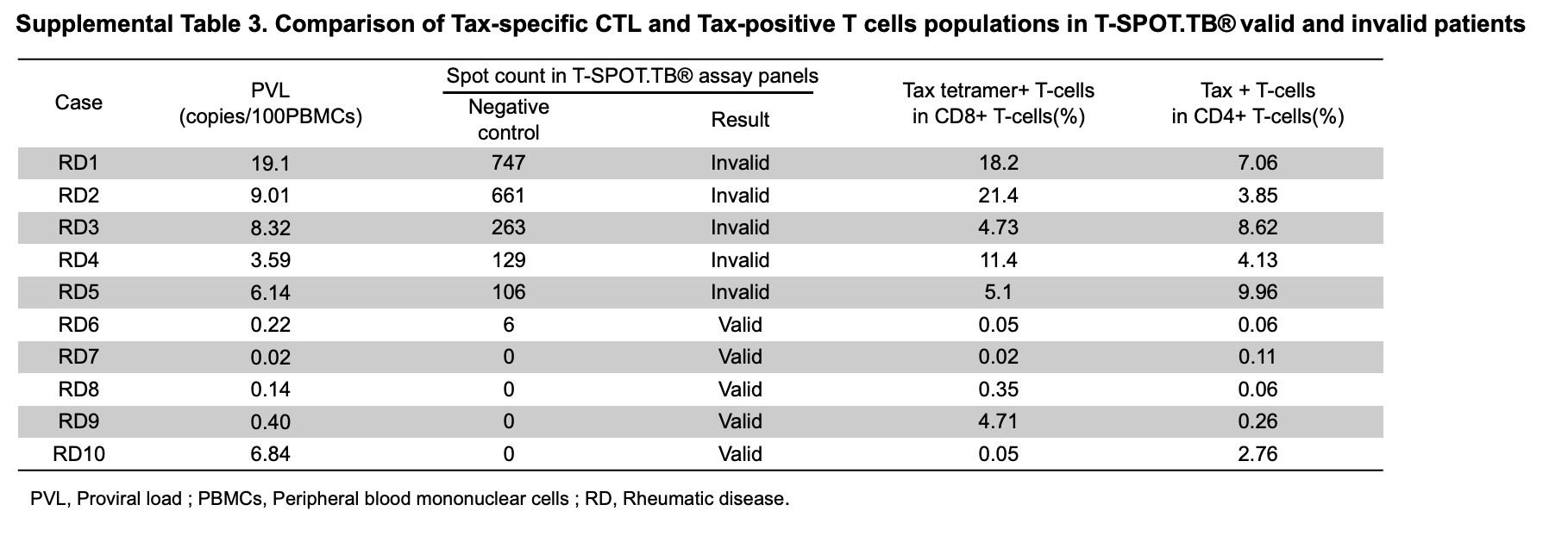

Supplement: Supplementary file 1 [file DataSheet1.docx]
